# A network autoregressive model with GARCH effects and its applications

**DOI:** 10.1371/journal.pone.0255422

**Published:** 2021-07-29

**Authors:** Shih-Feng Huang, Hsin-Han Chiang, Yu-Jun Lin

**Affiliations:** 1 Dept. Applied Mathematics, National University of Kaohsiung, Kaohsiung, Taiwan; 2 Institute of Statistics, National University of Kaohsiung, Kaohsiung, Taiwan; Feng Chia University, TAIWAN

## Abstract

In this study, a network autoregressive model with GARCH effects, denoted by NAR-GARCH, is proposed to depict the return dynamics of stock market indices. A GARCH filter is employed to marginally remove the GARCH effects of each index, and the NAR model with the Granger causality test and Pearson’s correlation test with sharp price movements is used to capture the joint effects caused by other indices with the most updated market information. The NAR-GARCH model is designed to depict the joint effects of nonsynchronous multiple time series in an easy-to-implement and effective way. The returns of 20 global stock indices from 2006 to 2020 are employed for our empirical investigation. The numerical results reveal that the NAR-GARCH model has satisfactory performance in both fitting and prediction for the 20 stock indices, especially when a market index has strong upward or downward movements.

## Introduction

**Fig 1:**
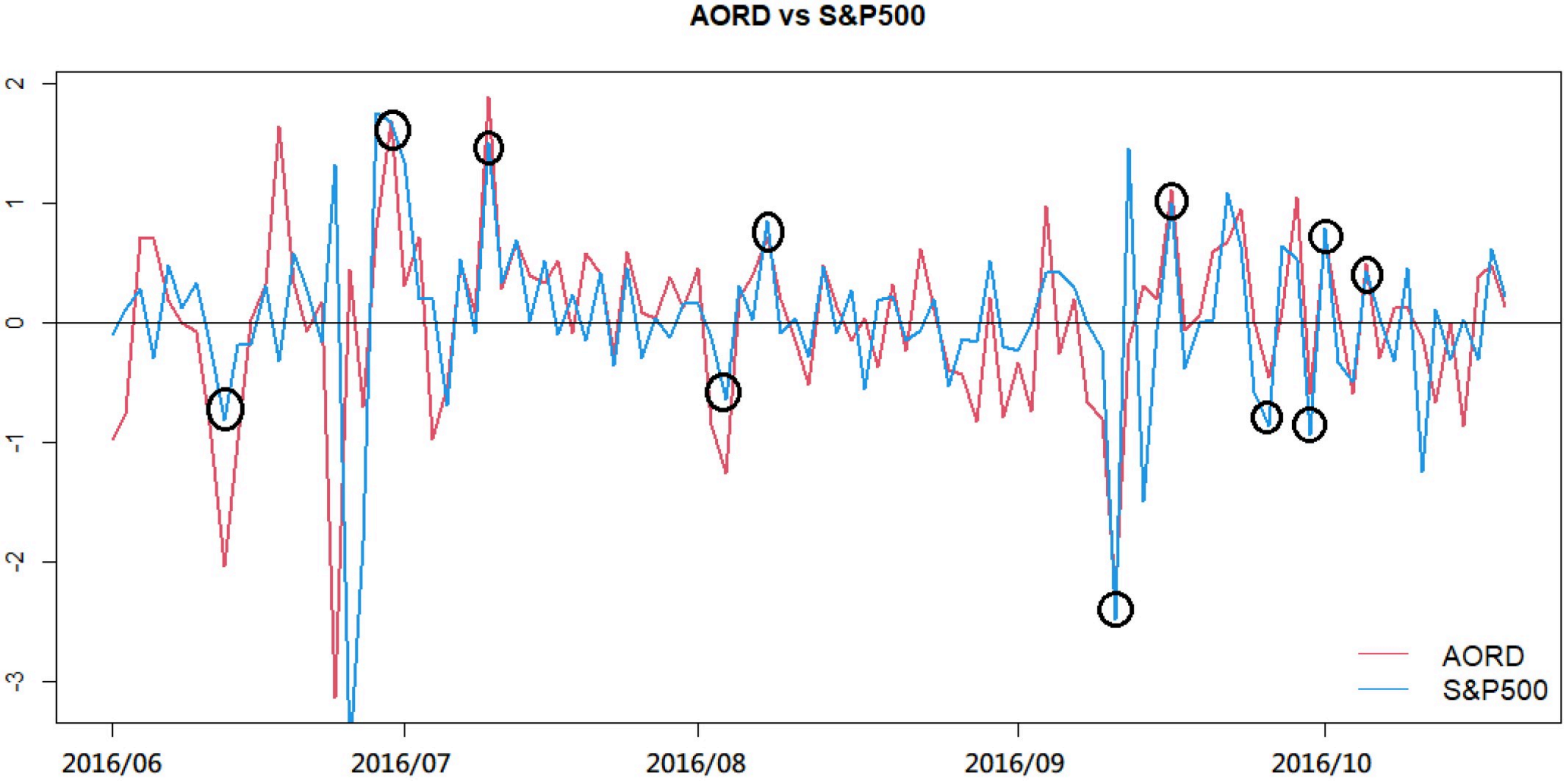
Log returns of the AORD and S&P500 indices from Jun. to Oct. in 2016.

The prediction of market trends has attracted much attention since the last century. Market participants make investment decisions according to their prediction of market trends. Thanks to the rapid development of information and communication technologies, market participants have opportunities to receive online information, such as the latest closing prices of global stock indices, updates on significant world events, and the newest economic policies announced by the most influential countries in the world. This real-time information has affected financial market trends, especially causing large shocks in stock indices. For example, Fig 1 presents an example to show the leading effect of the S&P500 index on the AORD index, which is Australia’s major index. In Fig 1, the red line denotes the returns of the AORD index from June 1, 2016, to Oct. 20, 2016, while the blue line presents the returns of the S&P500 from May 31, 2016, to Oct. 19, 2016, which are one day before the associated AORD trading dates. As shown in Fig 1, the two return processes have similar patterns, which indicates that the S&P 500 index returns did show their leading effects on the AORD returns, especially for most of those returns having large volatilities, during this specific time period. Usually, this type of relationship between different markets changes dynamically, which increases the difficulty of implementing the latest and helpful information into the timely prediction of market trends. Therefore, this study aims to analyze nonsynchronous multiple time series. An effective way is proposed to overcome the difficulty caused by the asynchronicity of major global indices for obtaining satisfactory predictions using the most updated information.

To achieve our objective, we have to address several challenges. The first task is to propose a model to describe the important features inherent in each stock index return and capture the joint effects among different return processes simultaneously. In addition, the proposed model needs to accommodate the most updated market information effectively. Moreover, the proposed model should be capable of avoiding the curse of dimensionality problems, and the estimation of the proposed model should not rely on a heavy computational burden. For the analysis of multivariate time series, the vector autoregression (VAR) model is a conventional method used by researchers and market participants (see [1–3] and the references therein). In addition, many empirical findings indicate that the return process of a stock index usually has the features of potential autocorrelation, conditional heteroscedasticity, volatility clustering, asymmetry, and heavy-tailed distribution. To characterize these features, GARCH-type models are commonly used in economics, finance, and statistics (see [1, 4–8] and the references therein). Therefore, to simultaneously consider the joint effects among multiple return processes and the above features contained in each financial time series, a VAR model with the de-GARCH technique is widely used for modeling multivariate time series (see [9–11] and the references therein). Another popular method is to adopt copula-based approaches to describe the dynamics of returns of multiple stock indices, where the marginal distribution of the returns of each index is characterized by a GARCH-type model, and the joint distribution of the returns of different indices are modeled by a copula function [12]. However, the computational costs of the above two approaches increase dramatically when the number of considered time series increases due to the curse of dimensionality.

To tackle these challenges, a network autoregressive (NAR) model of [13] with the de-GARCH technique, denoted by NAR-GARCH, is proposed in this study. Specifically, the NAR-GARCH model first filters out the GARCH effects contained in each return process. Next, an NAR model is employed to capture the joint effects among the de-GARCH processes, where a systematic scheme for accommodating the most updated market information is also proposed under the framework of the Granger causality test [14] and Pearson’s correlation test with sharp price movements. To investigate the performance of the proposed method, the daily index values of 20 stock markets, including AORD (Australia), BSESN (India), BVSP (Brazil), DAX (Germany), FCHI (France), FTSE (United Kingdom), GSPTSE (Canada), HSI (Hong Kong), JTOPI (South Africa), KLSE (Malaysia), KS11 (South Korea), MERV (Argentina), MXX (Mexico), N225 (Japan), RTSI (Russia), SSE (China), STI (Singapore), S&P500 (United States), TASI (Saudi Arabia), and XU100 (Turkey), from 2006 to 2020 are employed for our empirical study. The numerical results reveal that the NAR-GARCH model is capable of improving the prediction of market trends, especially when a market has sharp upward or downward movements. We also propose a trading strategy based on the prediction of market trends. The results indicate that the proposed trading strategy has better and more stable investment performances than the associated index value with transaction costs.

The remainder of this paper is organized as follows. Section 2 illustrates the reasons for how the proposed NAR-GARCH model is capable of describing the features of nonsynchronous multiple financial time series. Section 3 introduces an easy-to-implement procedure to estimate the proposed model. An empirical study based on 20 global stock indices is provided to investigate the performance of the NAR-GARCH model in Section 4. Discussions are presented in Section 5.

## Nonsynchronous multiple time series

To depict the dynamics of nonsynchronous multiple financial time series with GARCH effects, we propose to adopt a de-GARCH technique for removing the GARCH effects of each time series at first and capture the most updated cross effects by NAR models accordingly. In this section, we briefly illustrate a de-GARCH method based on fitting a GARCH model for each time series. The commonly used VAR models and the difficulty of using regular VAR models to handle nonsynchronous multiple financial time series are also discussed.

### DeGARCH method

Let $F_{t}$ denote the information set generated by the data up to time *t*. The following GARCH-type model is considered to be used for describing the dynamics of the return process of the *j*-th index, *j* = 1, …, *N*,

$$\begin{array}{c} \left\{ \begin{array}{ccc} r_{j,t} & = & \mu \left(r_{j,s},a_{j,s},s=t-1,t-2,\ldots ;\psi_{j}\right)+a_{j,t},\\ a_{j,t} & = & \sigma_{j,t}\varepsilon_{j,t},\\ \sigma_{j,t}^{2} & = & g(\sigma_{j,s},a_{j,s},s=t-1,t-2,\ldots ;\beta_{j}), \end{array}\right. \end{array}$$ (1)

where *r*_*j*,*t*_ denotes the log return of the *j*-th index at time *t*, *μ*(⋅) and *g*(⋅) are $F_{t-1}$-measurable functions, {*ε*_*j*,*t*_, *t* = 1, 2, …} is assumed to be a white noise process with zero mean and unit variance, and (***ψ***_*j*_, ***β***_*j*_) denotes the unknown parameters for the *j*-th process and satisfies the stationary conditions. This type of model (1) is capable of describing many important features of univariate financial time series, such as autocorrelation, conditional heteroscedasticity, volatility clustering and asymmetry, and is widely used by practitioners in the fields of economics, statistics and finance (see [1, 4, 5, 8, 15] and the references therein). For example, the autoregressive-moving-average (ARMA) structure is one of the most popular settings for *μ*(⋅). The classical GARCH model proposed by [4] and the EGARCH model of [5] are both commonly used setups for *g*(⋅).

Once the model (1) is employed to fit the return process of the *j*-th index, the corresponding de-GARCH return process, denoted by $\left\{{\tilde{r}}_{j,t},t=1,2,\ldots \right\}$, and standardized residual process, denoted by $\left\{{\hat{\varepsilon }}_{j,t},t=1,2,\ldots \right\}$, are obtained by

$$\begin{array}{c} {\tilde{r}}_{j,t}=\frac{r_{j,t}}{\sqrt{{\hat{g}}_{j,t}}}, \end{array}$$ (2)

and

$$\begin{array}{c} {\hat{\varepsilon }}_{j,t}=\frac{r_{j,t}-{\hat{\mu }}_{j,t}}{\sqrt{{\hat{g}}_{j,t}}}, \end{array}$$ (3)

respectively, where ${\hat{\mu }}_{j,t}=\mu \left(r_{j,s},{\hat{a}}_{j,s},s=t-1,t-2,\ldots ;{\hat{\psi }}_{j}\right)$ and ${\hat{g}}_{j,t}=g\left({\hat{\sigma }}_{j,s},{\hat{a}}_{j,s},s=t-1,t-2,\ldots ;\hat{\beta_{j}}\right)$, in which ${\hat{\psi }}_{j}$, ${\hat{\beta }}_{j}$, ${\hat{\sigma }}_{j,s}$ and ${\hat{a}}_{j,s}$ denote the estimates of ***ψ***_*j*_, ***β***_*j*_, *σ*_*j*,*s*_ and *a*_*j*,*s*_, respectively, and can be obtained under an iterative procedure with (1).

### VAR-GARCH model

As mentioned above, a VAR model with the de-GARCH technique is widely used for modeling multivariate time series. We briefly illustrate its procedure in the following. The first step is to obtain the de-GARCH returns, ${\tilde{r}}_{j,t}$, of the *j*-th index by (2), *j* = 1, …, *N*. Next, the following VAR model of order *p* is established for these ${\tilde{r}}_{j,t}$, *j* = 1, …, *N*, *t* = 1, …, *T*, processes:

$$\begin{array}{c} {\tilde{r}}_{t}=c+\sum_{k=1}^{p}A_{k}{\tilde{r}}_{t-k}+e_{t}, \end{array}$$ (4)

where ${\tilde{r}}_{t}={\left({\tilde{r}}_{1,t},\ldots ,{\tilde{r}}_{N,t}\right)}^{⊤}$ denotes the vector of de-GARCH returns of the *N* indices at time *t*, **c** is an *N*-dimensional constant vector, *A*_*k*_ is an *N* × *N* matrix and denotes the lag-*k* coefficient matrix associated with ${\tilde{r}}_{t-k}$, *k* = 1, …, *p*, and *e*_*t*_, *t* = 1, …, *T*, are i.i.d. *N*-dimensional Gaussian random vectors with a zero mean vector and covariance matrix Σ. Herein, we denote model (4) by VAR-GARCH. The parameter matrices **c** and *A*_*k*_, *k* = 1, …, *p*, in (4) can be estimated under the maximum likelihood or Bayesian framework. In this study, we adopted the R package ‘vars’ to estimate the VAR model. Let $\hat{c}$ and ${\hat{A}}_{k}$ denote the estimates of **c** and *A*_*k*_, respectively. According to (4), the one-step-ahead prediction of $r_{t+1}={\left(r_{1,t+1},\ldots ,r_{N,t+1}\right)}^{⊤}$ conditional on $F_{t}$, denoted by ${\hat{r}}_{t+1}={\left({\hat{r}}_{1,t+1},\ldots ,{\hat{r}}_{N,t+1}\right)}^{⊤}$, is obtained by

$$\begin{array}{c} {\hat{r}}_{t+1}=E\left(r_{t+1}|F_{t}\right)=(\hat{c}+\sum_{k=1}^{p}{\hat{A}}_{k}{\tilde{r}}_{t-k+1})\circ {\left({\hat{\sigma }}_{1,t+1},\ldots ,{\hat{\sigma }}_{N,t+1}\right)}^{⊤}, \end{array}$$ (5)

where *E*(*A*|*B*) denotes the conditional expectation of *A* given *B*, and the operator ‘∘’ denotes the Hadamard product.

However, a VAR model has substantial coefficient dimensionality, which would cause some computational problems for coefficient inference as *N* increases. To handle this problem, many VAR studies have recently focused on reducing the coefficient dimensionality via variable selection approaches based on some model-structure assumptions or adding sparsity conditions to the coefficient matrices (see [2, 3] and the references therein). Although these recently developed approaches do reduce coefficient dimensionality, the algorithms still require substantial computational time.

Another limitation of the VAR-GARCH model defined in (1)–(5) is that the model only considers the information from other indices up to the trading date *t* for obtaining a one-step-ahead prediction of **r**_*t*+1_ but cannot accommodate the most updated information from other indices within the trading date *t* + 1. For example, on Aug. 13, 2020, the closing price of the AORD index is already observed before the opening time of the S&P500 index. Hence, the closing price of the AORD index on Aug. 13, 2020, may provide helpful information for the prediction of the S&P500 index on the same trading date. Similarly, the closing price of the N225 index on Aug. 13, 2020, may also provide helpful information for the prediction of the S&P500 on the same date. However, this is not the case when using the AORD index for the prediction of N225. The most updated closing price of AORD for the prediction of N225 on Aug. 13, 2020, is observed on the previous trading date (Aug. 12, 2020). As shown in (4), to model the return processes of the AORD, N225, and S&P500 indices in a VAR model and make one-step-ahead predictions for the 3 indices on Aug. 13, 2020, one can only consider the closing prices of the 3 indices up to the previous trading date (Aug. 12, 2020). This limitation causes the regular VAR-GARCH model as defined in (4) to not accommodate the most updated information and may miss potentially helpful information that occurred in AORD and N225 on Aug. 13, 2020, for the prediction of the S&P500 on the same date. In other words, the VAR-GARCH model defined in (4) may miss potentially helpful information when modelling nonsynchronous multiple time series.

To deal with nonsynchronous multiple time series under the VAR-GARCH framework, one feasible approach is to redesign the coefficient matrices in (4) and add some constraints on the coefficient matrices. For example, consider the case of the AORD, N225 and S&P500 indices mentioned above, and let ${\tilde{r}}_{1,t}$, ${\tilde{r}}_{2,t}$ and ${\tilde{r}}_{3,t}$, respectively, denote their daily de-GARCH returns. Since the most updated information for ${\tilde{r}}_{1,t}$ and ${\tilde{r}}_{2,t}$ from the three market indices are ${\tilde{r}}_{i,t-1}$, *i* = 1, 2, 3, while the most updated information for ${\tilde{r}}_{3,t}$ from the three market indices are ${\tilde{r}}_{1,t}$, ${\tilde{r}}_{2,t}$ and ${\tilde{r}}_{3,t-1}$, a modified VAR-GARCH model of order 1 for reflecting these properties can be represented as

$$\begin{array}{ccc} \left(\begin{array}{c} {\tilde{r}}_{1,t}\\ {\tilde{r}}_{2,t}\\ {\tilde{r}}_{3,t} \end{array}\right) & = & \left(\begin{array}{c} c_{1}\\ c_{2}\\ c_{3} \end{array})+(\begin{array}{ccc} 0 & 0 & 0\\ 0 & 0 & 0\\ \beta_{31} & \beta_{32} & 0 \end{array})(\begin{array}{c} {\tilde{r}}_{1,t}\\ {\tilde{r}}_{2,t}\\ {\tilde{r}}_{3,t} \end{array}\right)\\ & + & \left(\begin{array}{ccc} \beta_{11} & \beta_{12} & \beta_{13}\\ \beta_{21} & \beta_{22} & \beta_{23}\\ 0 & 0 & \beta_{33} \end{array})(\begin{array}{c} {\tilde{r}}_{1,t-1}\\ {\tilde{r}}_{2,t-1}\\ {\tilde{r}}_{3,t-1} \end{array})+(\begin{array}{c} e_{1,t}\\ e_{2,t}\\ e_{3,t} \end{array}\right) \end{array}$$

where (*e*_1,*t*_, *e*_2,*t*_, *e*_3,*t*_)^⊤^, *t* = 1, 2, …, are i.i.d. random vectors as defined in (4). The above model is slightly different from the regular VAR-GARCH model of order 1 for modeling synchronous 3-dimensional time series. The estimation of this modified VAR-GARCH model and how to develop a suitable variable selection procedure for nonsynchronous multidimensional time series are in need of further study, which is beyond the scope of this research.

According to the above discussion, we aim to propose an alternative, easy-to-implement and effective procedure to avoid the estimation problem that occurs in the VAR model, especially when *N* is large.

## Methodology

To model nonsynchronous multiple time series with the most updated information in an easy-to-implement way, this study proposes to employ network models to describe the relationships between the nodes in a network cross time and accommodate the most updated information from other indices simultaneously. For example, a network model can be used to model the relationships between the users of Facebook or Twitter. In particular, the network autoregressive (NAR) model proposed by [13] is employed to describe the relationships between the returns of global stock indices in this study. Suppose that there are *N* nodes contained in a network and denote the value of the *j*-th node in a network at time *t* by *y*_*j*,*t*_, *j* = 1, …, *N* and *t* = 1, 2, …. The NAR model describes the dynamics of *y*_*j*,*t*_ by

$$\begin{array}{c} y_{j,t}=\beta_{j,0}+\sum_{\ell =1}^{p}\beta_{j,\ell }y_{j,t-\ell }+\sum_{k=1}^{q}\gamma_{j,k}n_{j}^{-1}\sum_{h=1}^{N}a_{j,h}y_{h,t-k}+Z_{j}^{⊤}\tau_{j}+\delta_{j,t}, \end{array}$$ (6)

where the term $\sum_{\ell =1}^{p}\beta_{j,\ell }y_{j,t-\ell }$ denotes the AR structure of order *p* for the {*y*_*j*,*t*_, *t* = 1, 2, …} process, *Z*_*j*_ is a *d*-dimensional covariate vector with *d* being a nonnegative integer, *τ*_*j*_ is a coefficient vector associated with *Z*_*j*_, $n_{j}^{-1}\sum_{h=1}^{N}a_{j,h}y_{h,t-k}$ represents the lag *k* network effect with *a*_*j*,*h*_ = 0 or 1 being the (*j*, *h*)-th component of an adjacency matrix, $n_{j}=\sum_{h=1}^{N}a_{j,h}$, and *δ*_*j*,*t*_’s are i.i.d. random variables with zero mean and finite variance $\sigma_{j}^{2}$ for *j* = 1, …, *N* and *t* = 1, 2, …. The model parameters, *β*_*j*,*ℓ*_, *ℓ* = 0, …, *p*, *γ*_*j*,*k*_, *k* = 1, …, *q*, *τ*_*j*_, and $\sigma_{j}^{2}$, *j* = 1, …, *N*, can be estimated by the classical least squares (LS) method. In [13], the asymptotic properties of the LS estimators are derived for the NAR model, and the numerical results reveal that the NAR model is capable of obtaining satisfactory performances.

The main advantage of the NAR model is that the autocorrelations and cross-correlations of the nodes in a network are included simultaneously in the model. In particular, the cross-correlations between the nodes are described by defining an adjacency matrix. However, despite these good properties, the NAR model cannot be applied directly to the modeling of the dynamics of global financial returns since the GARCH effects inherent in each return process have not yet been included. In addition, how to define a suitable adjacency matrix by a proper econometric method is still open. To address these situations, we propose extending the NAR model to incorporate GARCH effects and employ the Granger causality test and Pearson’s correlation test with sharp price movements to define a proper adjacency matrix. Most importantly, the adjacency matrix is established by accommodating the most updated closing prices of other indices. The details are illustrated in the following.

### NAR-GARCH model

In this section, we introduce the procedure of establishing the proposed NAR-GARCH model.

1. For each return process of each index, we employ the following ARMA(*p*_*j*_,*q*_*j*_)-GARCH: (${\tilde{p}}_{j}$,${\tilde{q}}_{j}$) model with standardized innovations being skewed-*t* distributed, which is a special case of model (1) and denoted by ARMA-GARCH-ST, to describe its dynamics:
  $$\begin{array}{c} \left\{ \begin{array}{ccc} r_{j,t} & = & \psi_{j,0}+\sum_{k=1}^{p_{j}}\psi_{j,k}r_{j,t-k}+a_{j,t}+\sum_{k=1}^{q_{j}}\psi_{j,p_{j}+k}a_{j,t-k}\\ a_{j,t} & = & \sigma_{j,t}\varepsilon_{j,t}\\ \sigma_{j,t}^{2} & = & \beta_{j,0}+\sum_{k=1}^{{\tilde{p}}_{j}}\beta_{j,k}a_{j,t-k}^{2}+\sum_{k=1}^{{\tilde{q}}_{j}}\beta_{j,{\tilde{p}}_{j}+k}\sigma_{j,t-k}^{2} \end{array},\right. \end{array}$$ (7)
  where *ε*_*t*_’s are i.i.d. skewed-*t* random variables with zero mean and unit variance, *p*_*j*_, *q*_*j*_, ${\tilde{p}}_{j}$ and ${\tilde{q}}_{j}$ are nonnegative integers, and $\psi_{j}=\left(\psi_{j,0},\ldots ,\psi_{j,p_{j}+q_{j}}\right)$ and $\beta_{j}=\left(\beta_{j,0},\ldots ,\beta_{j,{\tilde{p}}_{j}+{\tilde{q}}_{j}}\right)$ both satisfy stationary conditions.
  In this study, we adopt the *R* package ‘garchFit’ to estimate the parameters in an ARMA-GARCH-ST model, where the probability density function of the skewed-*t* distribution is defined by
  $$\begin{array}{c} f\left(y\right)=C{\left\{1+{\left(y-\mu \right)}^{2}/\left(\nu \sigma^{2}\right)\left[1/\gamma^{2}I\left(y\geq \mu \right)+\gamma^{2}I\left(y<\mu \right)\right]\right\}}^{-(\nu +1)/2} \end{array}$$ (8)
  with location *μ*, scale *σ*, degrees of freedom *ν*, skewness parameter *γ*, and a positive constant *C* such that $\int_{-\infty }^{\infty }f\left(y\right)dy=1$ [16].
  The ARMA-GARCH-ST model has been shown to be capable of depicting the dynamics of financial time series well in the literature [8, 17–19]. In particular, the skewed-*t* distribution defined in (8) is a special case of the skewed generalized *t* distribution, denoted as SGT, which was proposed by [20], with a height parameter of 2. Nevertheless, the empirical results in [19] reveal that the estimated height parameters of SGT distributions under a rolling-window framework are quite stable at approximately 2. Therefore, the *R* package ‘garchFit’ is a suitable choice in practical implementation when fitting an ARMA-GARCH-ST model.
  Moreover, the orders $\left(p_{j},q_{j},{\tilde{p}}_{j},{\tilde{q}}_{j}\right)$ can be determined by employing an information criterion such as AIC or BIC. Consequently, the corresponding standardized residuals are obtained via (3) with
  $${\hat{\mu }}_{j,t}={\hat{\psi }}_{j,0}+\sum_{k=1}^{p_{j}}{\hat{\psi }}_{j,k}r_{j,t-k}+\sum_{k=1}^{q_{j}}{\hat{\psi }}_{j,p_{j}+k}{\hat{a}}_{j,t-k}$$
  and
  $${\hat{g}}_{j,t}={\hat{\beta }}_{j,0}+\sum_{k=1}^{{\tilde{p}}_{j}}{\hat{\beta }}_{j,k}{\hat{a}}_{j,t-k}^{2}+\sum_{k=1}^{{\tilde{q}}_{j}}{\hat{\beta }}_{j,{\tilde{p}}_{j}+k}{\hat{\sigma }}_{j,t-k}^{2}.$$
2. Fit the following NAR model for the standardized residuals obtained in Step 1:
  $$\begin{array}{c} {\hat{\varepsilon }}_{j,t}=\sum_{k=1}^{Q}\{\gamma_{j,k}n_{j,k}^{-1}\sum_{h=1}^{N}c_{j,h}^{\left(k\right)}{\hat{\varepsilon }}_{h,t-d_{h}^{j}-k+1}\}+\delta_{j,t}, \end{array}$$ (9)
  where *Q* is a predetermined integer, $n_{j,k}=\sum_{h=1}^{N}\left|c_{j,h}^{\left(k\right)}\right|$ with $c_{j,h}^{\left(k\right)}=0$, 1, or -1 being the (*j*, *h*)-th component of a lag-*k* adjacency matrix, which is defined by (10) below, and *δ*_*j*,*t*_’s are i.i.d. random variables with mean zero and variance $\sigma_{\delta }^{2}$. In addition, set $d_{h}^{j}=0$ if the closing time of the *h*-th index is earlier than the opening time of the *j*-th index at date *t*; $d_{h}^{j}=1$, otherwise. Moreover, ${\hat{\varepsilon }}_{h,t-d_{h}^{j}-k+1}$ denotes the corresponding lag-*k* standardized residual of the *h*-th index for the *j*-th index at date *t* with the definition of $d_{h}^{j}$.

The NAR model defined in (9) is a special case of model (6). Since the standardized residual process of each index obtained in Step 1 has a mean of zero and does not have autocorrelation, we remove the first two terms on the right-hand side (RHS) of (6). The 4th term on the RHS of (6) is also removed since we did not include any covariate yet in this study. In particular, if $d_{h}^{j}=0$ and *k* = 1, we have ${\hat{\varepsilon }}_{h,t-d_{h}^{j}-k+1}={\hat{\varepsilon }}_{h,t}$, which allows us to reflect the most updated closing prices of the *h*-th index for the *j*-th index within the same trading date *t*.

It is worth mentioning that the parameter *Q* in (9) represents the upper bound of possibly helpful information from other markets for the *j*-th index. From the perspective of the efficient market hypothesis, the index value would reflect all information very quickly. Since the indices considered in this study represent global markets and have high liquidity, *Q* should not be too large. Therefore, we set *Q* = 2, which means that we collect possibly helpful information from the last 2 trading days of other markets for a target index.

Next, we propose to adopt the Granger causality test and check whether the *h*-th index would cause a sharp price movement of the *j*-th index to determine the value of $c_{j,h}^{\left(k\right)}$ in the lag-*k* adjacency matrix in model (9). For example, to evaluate whether the *h*-th index has casual influence on the *j*-th index, *h* ≠ *j*, and if $d_{h}^{j}=0$ for this pair of indices, we consider the following regression,

$$\begin{array}{c} {\hat{\varepsilon }}_{j,t}=\sum_{k=1}^{Q}\alpha_{j,k}{\hat{\varepsilon }}_{h,t-k+1}+\zeta_{t}, \end{array}$$ (10)

where *Q* is defined the same as in (9) and *ζ*_*t*_’s are i.i.d. random variables with mean zero and variance $\sigma_{\zeta }^{2}$. Since ${\hat{\varepsilon }}_{j,t}’\text{s}$ and ${\hat{\varepsilon }}_{h,t}’\text{s}$ are estimated standardized residuals of the *j*- and *h*-th indices, respectively, they both have zero expectations, and thus, we do not have a constant term on the RHS of (10). In addition, since ${\hat{\varepsilon }}_{j,t}\text{s}$ should not have autocorrelations, we do not consider autoregressive terms on the RHS of (10) and set $c_{j,j}^{\left(k\right)}=0$ in (9). In view of (10), if there exists at least one *α*_*j*,*k*_ ≠ 0 significantly, *k* ∈ {1, …, *Q*}, we conclude that the *h*-th index has a causal influence on the *j*-th index. Furthermore, let *ρ*_*j*,*h*_(*A*_*j*_) denote the conditional correlation of the returns between the *j*-th and *h*-th indices given the event

$$\begin{array}{c} A_{j}=\left\{r_{j,s}<q\left(\tau \right)\;\text{or}\;r_{j,s}>q\left(1-\tau \right)\right\} \end{array}$$ (11)

with *τ* = 0.2, for instance, where *q*(*τ*) denotes the *τ*-th quantile of the distribution of {*r*_*j*,*s*_, *s* = *t* − 249, …, *t*}. If *α*_*j*,*k*_ and *ρ*_*j*,*h*_(*A*_*j*_) are both significantly nonzero for *j* ≠ *h*, set $c_{j,h}^{\left(k\right)}=\text{sign}\left({\hat{\rho }}_{j,h}\left(A_{j}\right)\right)$; otherwise, set $c_{j,h}^{\left(k\right)}=0$. The performance of this setting is thoroughly investigated in our empirical study.

According to (7)–(11), the proposed one-step-ahead prediction of **r**_*t*+1_, denoted by ${\hat{r}}_{t+1}^{*}={\left({\hat{r}}_{1,t+1}^{*},\ldots ,{\hat{r}}_{N,t+1}^{*}\right)}^{⊤}$, is obtained by

$$\begin{array}{c} {\hat{r}}_{t+1}^{*}=E\left(r_{t+1}|F_{t+1}^{*}\right)={\hat{\mu }}_{t+1}+{\hat{\sigma }}_{t+1}\circ {\hat{\varepsilon }}_{t+1}, \end{array}$$ (12)

where $F_{t+1}^{*}$ denotes the set containing the most updated information up to date *t* + 1, ${\hat{\mu }}_{t+1}={\left({\hat{\mu }}_{1,t+1},\ldots ,{\hat{\mu }}_{N,t+1}\right)}^{⊤}$ and ${\hat{\sigma }}_{t+1}={\left({\hat{\sigma }}_{1,t+1},\ldots ,{\hat{\sigma }}_{N,t+1}\right)}^{⊤}$ are both $F_{t}$-measurable and can be obtained from (7) recursively, and ${\hat{\varepsilon }}_{t+1}={\left({\hat{\varepsilon }}_{1,t+1},\ldots ,{\tilde{\varepsilon }}_{N,t+1}\right)}^{⊤}$ with

$${\hat{\varepsilon }}_{j,t+1}=\sum_{k=1}^{Q}\{{\hat{\gamma }}_{j,k}n_{j,k}^{-1}\sum_{h=1}^{N}c_{j,h}^{\left(k\right)}{\hat{\varepsilon }}_{h,t-d_{h}^{j}-k+2}\},$$

which is $F_{t+1}^{*}$-measurable since for any fixed *j* ∈ {1, …, *N*} and *k* ∈ {1, …, *Q*}, $c_{j,h}^{\left(k\right)}$ and ${\hat{\varepsilon }}_{h,t-d_{h}^{j}-k+2}$ are determined prior to the opening time of the *j*-th index on date *t* + 1 if $d_{h}^{j}=0$ for some *h* ∈ {1, …, *N*}.

The most different part for the one-step-ahead prediction of **r**_*t*+1_ via (5) or (12) is that the two models use different information sets. Intuitively, the proposed ${\hat{r}}_{t+1}^{*}$ defined in (12) should have better performance than the ${\hat{r}}_{t+1}$ defined in (5) since $F_{t}\subseteq F_{t+1}^{*}$, that is, $F_{t+1}^{*}$ contains more updated information than $F_{t}$. To investigate this conjecture, we provide an extensive empirical study in the next section.

### NAR-GARCH vs. VAR-GARCH

Since both the NAR-GARCH and VAR-GARCH models need to pass the GARCH filter in the first step, we compare their computational burden after de-GARCHing. For the VAR-GARCH model, the number of parameters for *N*-dimensional time series contained in **c** and *A*_*k*_ on the RHS of (4) is *N* + *pN*^2^. For the NAR-GARCH model, since the number of parameters for the *j*-th index, *j* = 1, …, *N*, on the RHS of (9) is *Q* + *N* − 1 because $c_{jj}^{\left(k\right)}=0$, the total number of parameters for the *N*-dimensional time series is (*Q* − 1)*N* + *N*^2^. In practice, the lags *p* and *Q* in VAR-GARCH and NAR-GARCH, respectively, would be small due to the efficient market hypothesis. In particular, if *p* = *Q* > 0, the NAR-GARCH model has fewer parameters than the VAR-GARCH model for large *N*. In addition, some computational challenges of the VAR-GARCH model have been mentioned in the previous sections. We focus on the details of the computational complexity of the NAR-GARCH model in the following.

Although the number of parameters still has the order of *O*(*N*^2^) in the NAR-GARCH model, the proposed estimation procedure can avoid the computational problem that occurred in the VAR-GARCH model. The main reason is that most of the parameters in (9) are contained in the adjacency matrix, where the components can be estimated pairwise. This study employs the Granger causality test and Pearson’s correlation test for each pair of time series to estimate the associated component in the adjacency matrix. These two tests can be done quickly by existing packages and do not require a heavy computational burden. Once the adjacency matrix is obtained, the remaining *Q* coefficients, *γ*_*j*,*k*_, in (9) are estimated by the commonly used LS method. In our empirical study with *N* = 20, the computational time of constructing an NAR-GARCH model for an index with 250 returns is approximately 1.7 seconds on a personal PC with an i7-10875H CPU and 8 GB RAM. Hence, the NAR-GARCH model can effectively capture the joint effects caused by other indices with the most updated market information.

Another advantage of the NAR-GARCH model is its high elasticity when including/excluding any index in/from the model. For example, suppose that one already established an NAR-GARCH model for *N* indices and he/she suddenly plans to include one more index in the model. In that case, he/she only needs to add one more row and column to update the adjacency matrix, where the components of the new row can be obtained by performing the Granger causality test and Pearson’s correlation test on the new index with each of the previous *N* indices sequentially. The new column is the transpose of the new row. Next, update the regression coefficients in (9) with the new adjacency matrix. In other words, only *Q* + *N* coefficients need to be re-estimated. Suppose he/she wants to exclude one index considered in the model. In that case, he/she only needs to delete the associated row and column from the original adjacency matrix and update the regression coefficients accordingly. Hence, only the *Q* regression coefficients in (9) need to be re-estimated. However, in the regular VAR-GARCH framework, every coefficient in (4) must be re-estimated when a user decides to include/exclude any index in/from the system. Therefore, the NAR-GARCH model has better elasticity and better accommodates re-estimation when including/excluding any index in/from the model compared to a VAR model.

## Empirical study

In this section, we investigate the fitting and prediction performances of the proposed NAR-GARCH model. The classical AR and ARMA-GARCH models and the VAR-GARCH model defined in (4) are employed for comparison. Based on the prediction of market trends from different methods, a trading strategy is proposed to investigate the corresponding investment performances.

### Prediction of market trends

The daily index values of 20 stock markets mentioned in Section 1 from 2006 to 2020 are employed for our empirical study. The data were downloaded from Yahoo Finance (https://finance.yahoo.com/) and Investing.com (https://www.investing.com/). A rolling-window approach with a window size of 250 trading days is used for model fitting. For each index, we employ model (7) with ${\tilde{p}}_{j}=1$ and *p*_*j*_, *q*_*j*_, and ${\tilde{q}}_{j}\in \left\{0,1\right\}$ for the returns in every rolling time interval. The model with the smallest AIC value is selected, and the corresponding standardized residuals are obtained. Next, we apply the Granger causality test to the standardized residuals of each pair of the 20 indices as in (10) with *Q* = 2, and apply Pearson’s correlation test to the event defined in (11) for the construction of lag-*k* adjacency matrices, *k* = 1, 2, with $c_{j,h}^{\left(k\right)}$ being the (*j*, *h*)-component in (9). Finally, we fit an NAR model defined in (9) for the standardized residuals with the two adjacency matrices, and the one-step-ahead predictions for the 20 indices computed by the proposed NAR-GARCH model are obtained by (12).

For comparison, we fit AR, ARMA-GARCH, and VAR-GARCH models separately for each index return. Among these 4 models, the AR model and ARMA-GARCH model are classical time series models, but the AR model does not consider the GARCH effects. Moreover, neither model captures the cross-correlations between indices. On the other hand, the VAR-GARCH takes the cross-correlations between indices into consideration, but it cannot accommodate the most updated information, as mentioned previously. Our objective is to investigate whether the cross-correlations and the most updated information described by the proposed NAR-GARCH model are capable of improving the prediction of market trends.

To evaluate the fitting performance, the following 3 types of measurements are applied to each return process in every rolling time interval (denoted by *r*_*t*_, *t* = 1, …, 250, for simplicity):

1. Mean squared error (MSE):
  $$\text{MSE}=\frac{1}{250}\sum_{t=1}^{250}{\left(r_{t}-{\hat{r}}_{t}\right)}^{2};$$
2. Mean absolute error (MAE):
  $$\text{MAE}=\frac{1}{250}\sum_{t=1}^{250}\left|r_{t}-{\hat{r}}_{t}\right|;$$
3. Partial mean squared error (PMSE):
  $$\text{PMSE}\left(\alpha \right)=\frac{1}{\sum_{t=1}^{250}I_{\left\{r_{t}<q\left(\alpha \right)\;\text{or}\;r_{t}>q\left(1-\alpha \right)\right\}}}\sum_{t=1}^{250}{\left(r_{t}-{\hat{r}}_{t}\right)}^{2}I_{\left\{r_{t}<q\left(\alpha \right)\;\text{or}\;r_{t}>q\left(1-\alpha \right)\right\}},$$

where ${\hat{r}}_{t}$ denotes the estimate of *r*_*t*_, *I*_*A*_ denotes the indicator function of an event *A*, *q*(*α*) denotes the *α*-th quantile of the distribution of {*r*_*s*_, *s* = 1, …, 250}, and *α* ∈ (0, 0.5).

Similarly, to evaluate the prediction performance, the ${\hat{r}}_{t}$ in the above 3 measurements denotes the one-step-ahead prediction of *r*_*t*_ and is estimated from the information set $F_{t-1}$ for the AR, GARCH, and VAR-GARCH models and from $F_{t}^{*}$ defined in (12) for the NAR-GARCH model. In addition, the *q*(*α*) at time *t* also turns to be estimated from the empirical distribution of the historical data {*r*_*s*_, *s* = *t* − 1, …, *t* − 249} for each index. To simplify the illustration, we did not use different notations for the 3 measurements when evaluating fitting or prediction performances.

The MSE and MAE are two widely used measurements to evaluate model performance, while the PMSE(*α*) with a small *α* is adopted to evaluate the performance when *r*_*t*_ is highly volatile. In principle, if a return process is highly volatile during a time interval, it means that the return process has a larger risk and the prediction of the trend of the process plays a more important role than a less volatile time period.

**Fig 2:**
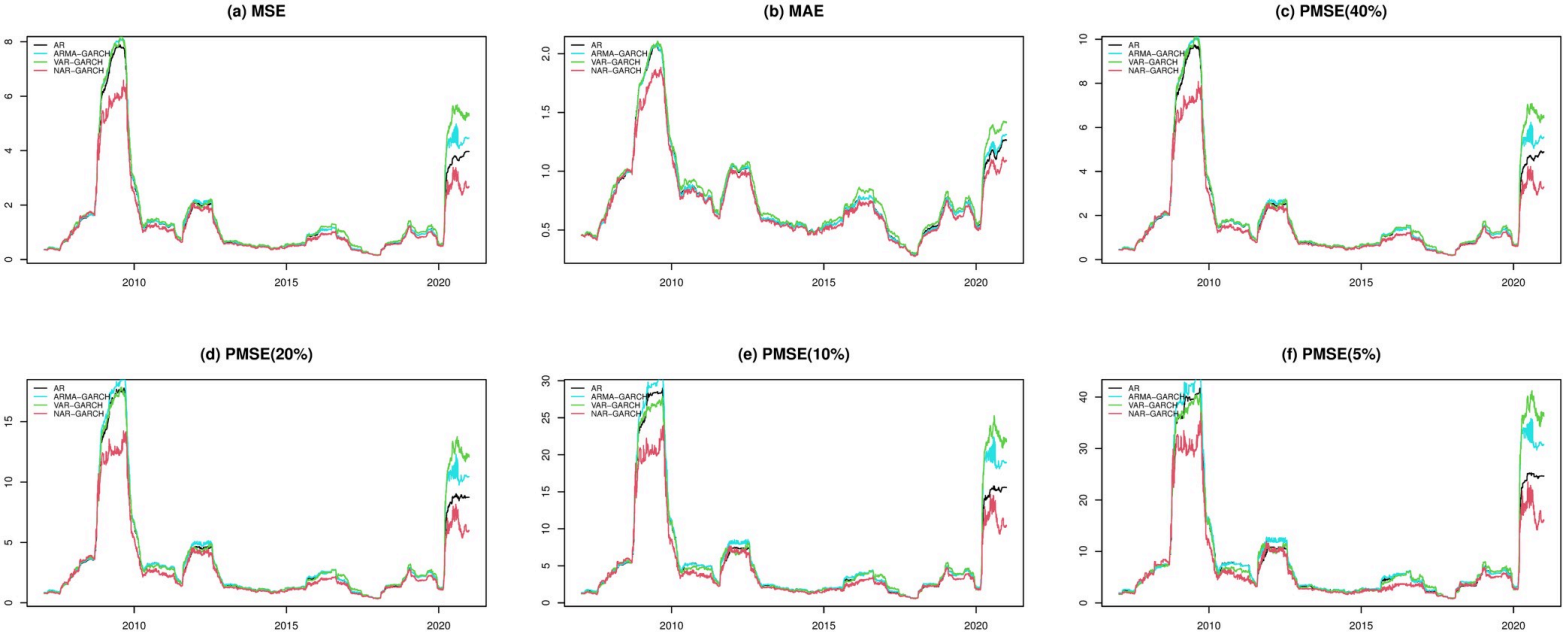
The fitted performances of the 4 models for the S&P 500 index.

**Fig 3:**
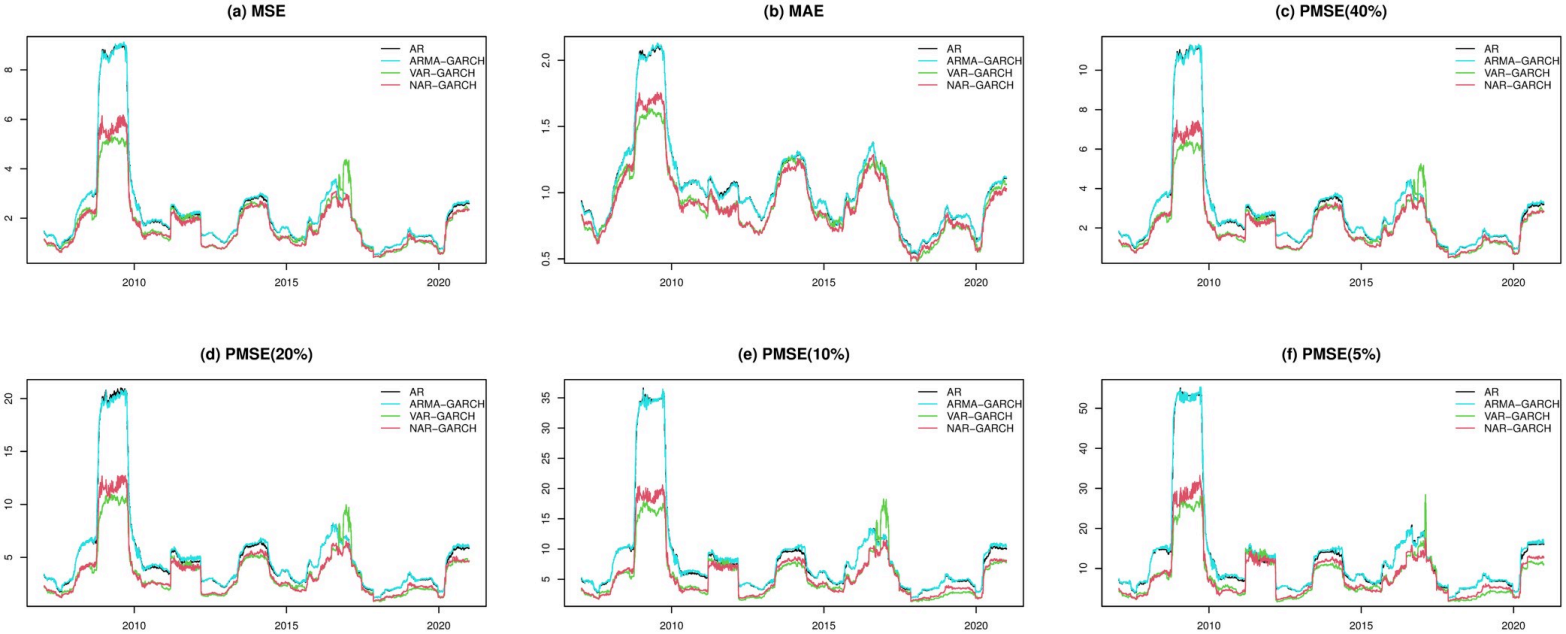
The fitted performances of the 4 models for the N225 index.

**Fig 4:**
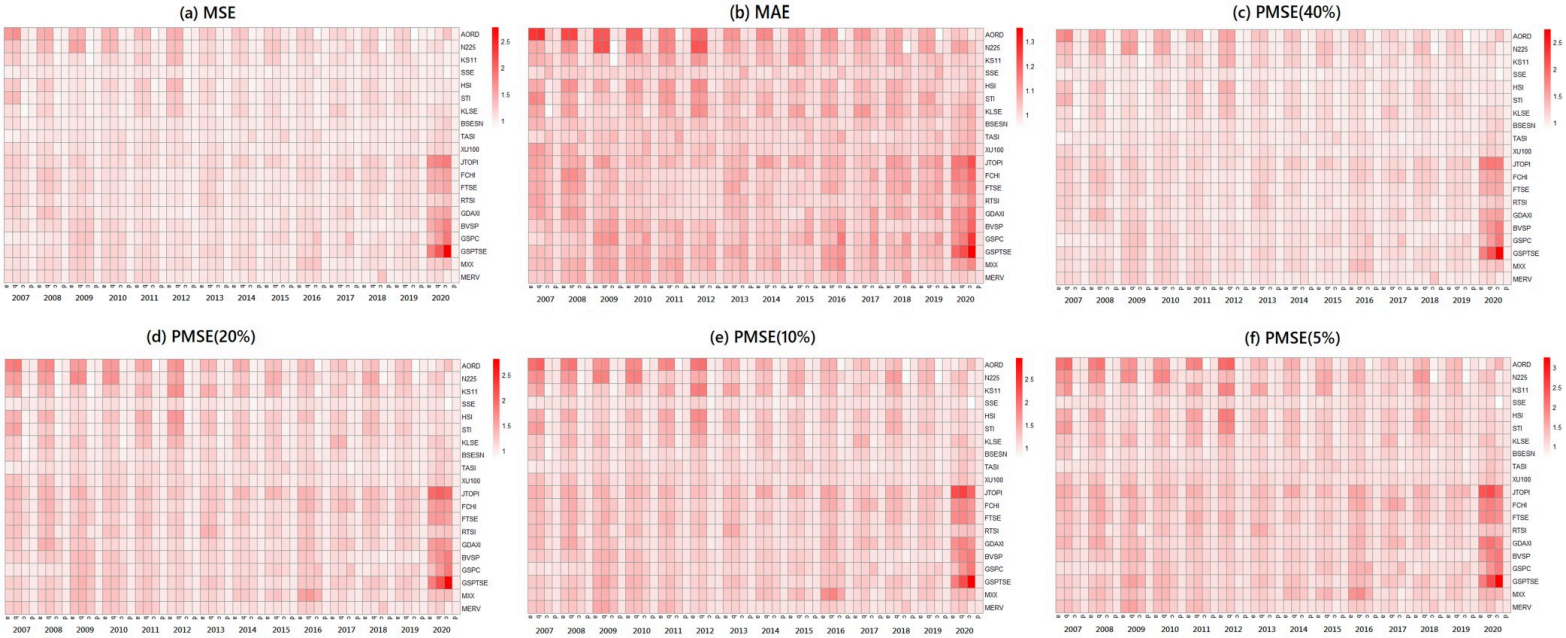
The fitted performances of the 4 models for the 20 indices from 2007 to 2020.

In Figs 2 and 3, we present the fitted performances of the 4 models for the S&P 500 index and N225 index, respectively, where the lag parameter *Q* in the step of the Granger causality test is set to 2. In both figures, the NAR-GARCH model has smaller values among the 6 measurements (MSE, MAE, and PMSE(*α*) with *α* = 0.05, 0.1, 0.2 and 0.4) than the others for most time periods, especially during the time periods of financial crisis in 2008-2009 and COVID-19 in 2020. In particular, VAR-GARCH had the best performance for the N225 index in 2008-2009. Furthermore, we present heatmaps of the 6 measurements of the 20 indices annually during the investigation time period in Fig 4, where the y-axis lists the codes of the 20 indices and the x-axis lists the years. In each year, the measurements of the AR, ARMA-GARCH, VAR-GARCH, and NAR-GARCH are presented sequentially. One can find that the NAR-GARCH model has the most stable and smallest values of the 6 measurements for each index during 2007-2020. In particular, comparing the values of PMSE(*α*) with different *α*, one can find that the AR and ARMA-GARCH models perform significantly more poorly than the VAR-GARCH and NAR-GARCH models when *α* decreases since the darkening rates of the colors of the AR and ARMA-GARCH models are more significant than the other 2 models from Fig 4(c) to 4(f). This finding highlights the importance and advantages of modeling the cross-correlation among different indices. From Figs 2–4, we conclude that the VAR-GARCH and NAR-GARCH models have better fitting performances than the AR and ARMA-GARCH models for the 20 indices during 2007-2020.

**Fig 5:**
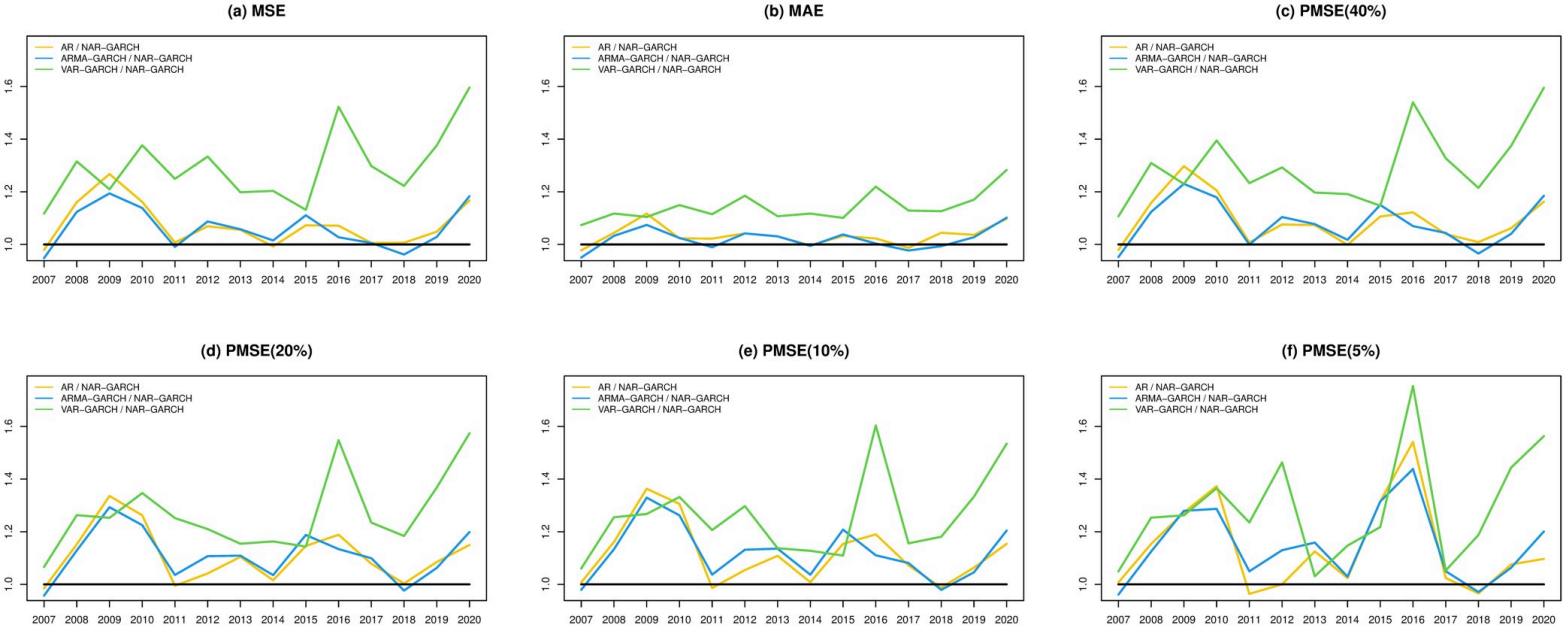
The prediction performances of the 4 models for the S&P 500 index.

**Fig 6:**
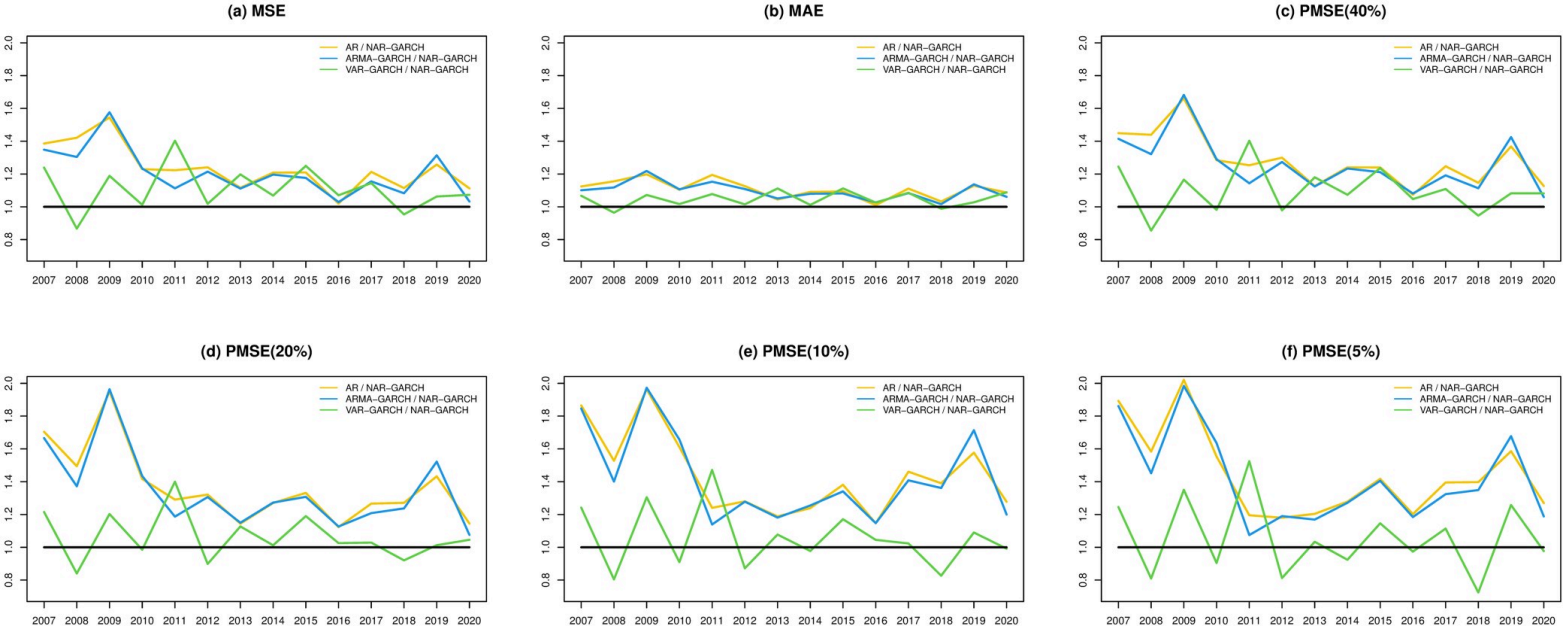
The prediction performances of the 4 models for the N225 index.

**Fig 7:**
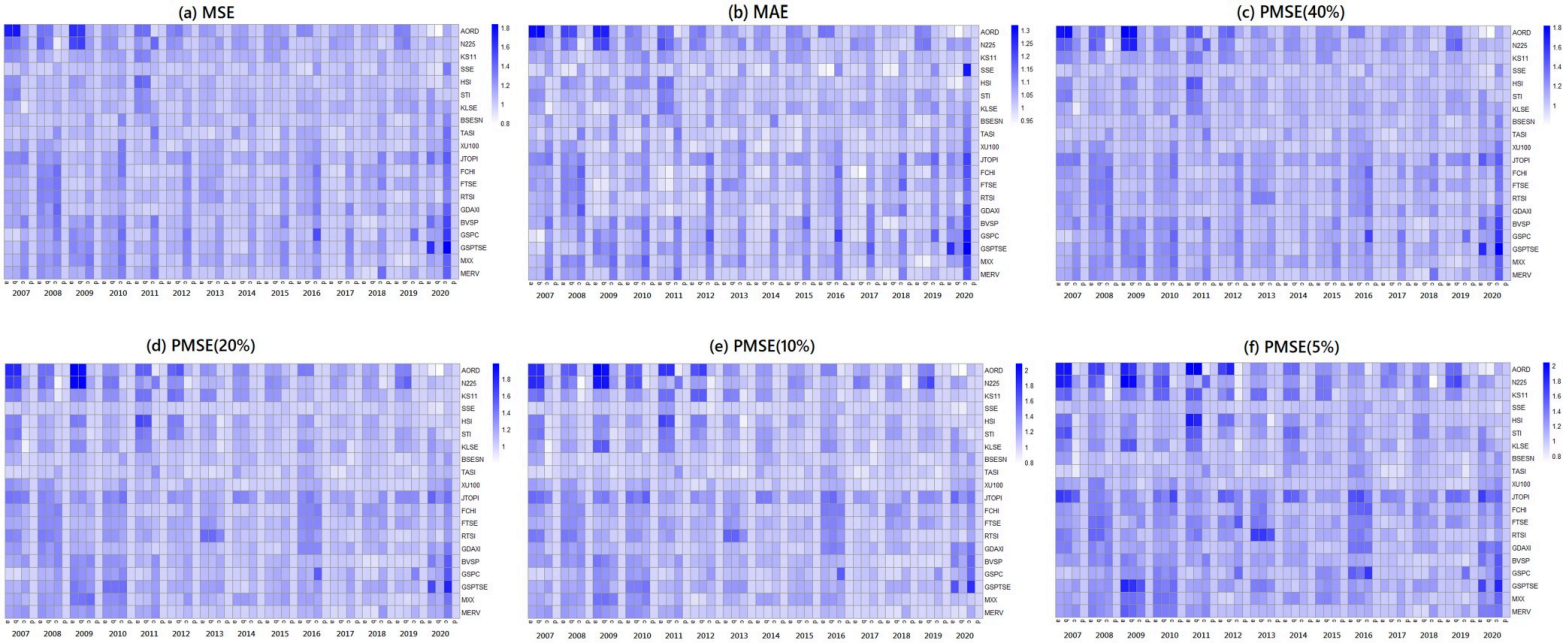
The prediction performances of the 4 models for the 20 indices from 2007 to 2020.

Next, we investigate the prediction performances of the 4 models. Similar to Figs 2–4, we compute the 6 measurements of the one-step-ahead predictions of the 4 models. The results are presented in Figs 5–7. In particular, we additionally divide the measurement values of the AR, ARMA-GARCH, and VAR-GARCH models by the associated values of the NAR-GARCH model in Figs 5 and 6. The results in Figs 5–7 reveal that the NAR-GARCH model has the most robust and better prediction performances than the other 3 models. Accordingly, we find that the NAR-GARCH model is capable of improving the prediction of market trends, especially when a market has strong upward or downward movements. In the next section, we conduct an investment strategy based on market trend predictions and investigate whether accurate trend prediction could remarkably increase investment performance.

### Investment performance

In this section, we propose the following trading strategy based on the market trend predictions from the 4 models and compare their investment performances. Let ${\hat{r}}_{t}$ denote the one-step-ahead prediction of *r*_*t*_, which is defined the same as in the previous section. Initially, set *t* = 1, and perform the following procedure with a predetermined *α* ∈ (0, 0.5).

1. Before the opening time of the market on date *t*, if ${\hat{r}}_{t}>q\left(1-\alpha \right)$, set a long position of the index; if ${\hat{r}}_{t}<q\left(\alpha \right)$, set a short position of the index; otherwise, do nothing at the opening time of the market on date *t*.
2. Close the position at the closing time on date *t*.
3. Let *t* = *t* + 1, and repeat the above 2 steps until the end of the investigation time period.

Let $P_{t}^{0}$ and $P_{t}^{1}$ denote the opening and closing values, respectively, of the underlying index on date *t*. Similarly, let $V_{t}^{0}$ and $V_{t}^{1}$ denote the opening and closing values, respectively, of the portfolio on date *t*. In addition, let *V*(*t*) = *k*_*c*_ *P*(*t*), which means that, for example, 1 point of the S&P500 index costs *k*_*c*_ US dollars. To consider transaction costs when performing the above trading strategy in each year and to simplify the illustration, let *t*_*j*_, *j* = 1, …, *m* denote the dates when setting a long or short position based on the above trading strategy with market predictions in a year. In particular, let *k*_*p*_ denote the commission rate proportional to the value of a portfolio, *k*_*f*_ denote a fixed amount of commission for trading the underlying index per share, and *k*_*t*_ denote the tax or other expenses when selling the underlying index per share.

In the following investigation, we consider a long or short 1 share of an underlying index at the opening time and close the position at the closing time on date *t*_*j*_, and we denote the associated profit by Δ*V*(*t*_*j*_). For simplicity, we ignore the effect of the interest rate. Therefore, the annual investment profit is computed by

$$\begin{array}{c} \Delta V=\sum_{j=1}^{m}\Delta V\left(t_{j}\right). \end{array}$$ (13)

Specifically, if the underlying index is S&P500, the profit of setting a long position on date *t*_*j*_ and closing it on the same trading date is

$$\Delta V\left(t_{j}\right)=k_{c}\left(P_{t_{j}}^{1}-P_{t_{j}}^{0}\right)-\left(k_{c}P_{t_{j}}^{1}\times k_{p}+2k_{f}+k_{t}\right),$$

and the profit of setting a short position on date *t*_*j*_ and closing it on the same day is

$$\Delta V\left(t_{j}\right)=k_{c}\left(P_{t_{j}}^{0}-P_{t_{j}}^{1}\right)-\left(k_{c}P_{t_{j}}^{0}\times k_{p}+2k_{f}+k_{t}\right).$$

If the underlying index is N225, the profit of setting a long position on date *t*_*j*_ and closing it on the same day is

$$\Delta V\left(t_{j}\right)=k_{c}\left\{P_{t_{j}}^{1}\left(1-k_{p}\right)-P_{t_{j}}^{0}\left(1+k_{p}\right)\right\},$$

and the profit of setting a short position on date *t*_*j*_ and closing it on the same day is

$$\Delta V\left(t_{j}\right)=k_{c}\left\{P_{t_{j}}^{0}\left(1-k_{p}\right)-P_{t_{j}}^{1}\left(1+k_{p}\right)\right\}.$$

**Fig 8:**
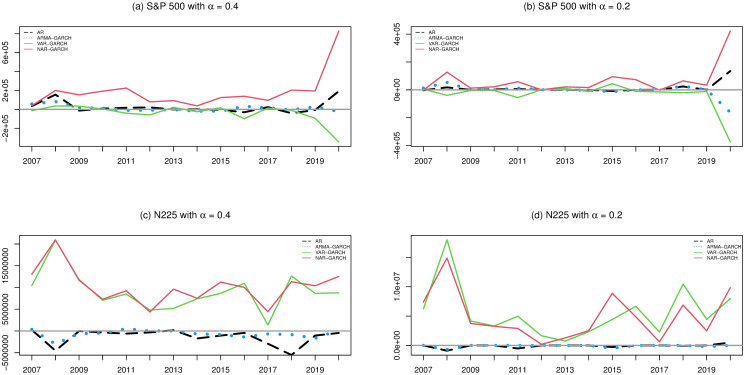
The investment performances of the 4 models for the S&P 500 and N225 indices from 2007 to 2020 with *α* = 0.2 and 0.4.

Fig 8 presents Δ*V* defined in (13) of each market trend prediction model during 2007-2020 for the S&P500 and N225 indices with *α* = 0.2 and 0.4 under the assumption of $P_{t}^{0}=P_{t-1}^{1}$, where we set (*k*_*c*_, *k*_*p*_, *k*_*f*_, *k*_*t*_) = (250, 0.00221%, 0.003, 0.000119) for the S&P500 index, and (*k*_*c*_, *k*_*p*_, *k*_*f*_, *k*_*t*_) = (1000, 0.0822%, 0, 0) for the N225 index. From Fig 8, one can find that the NAR-GARCH model has the best investment performances in most cases according to its accurate market trend predictions. In particular, when a market has large upward or downward movements in an investigation year (for example, 2008 and 2020), the numerical results reveal significant superiority of the investment profit based on the NAR-GARCH model over the other 3 competitors in these two markets. This phenomenon highlights that market participants could benefit from obtaining accurate market trend predictions of large movements by accommodating the most updated information via the proposed approach.

## Discussion

In this study, we propose an NAR-GARCH model to describe the important features inherent in each stock index return and capture the joint effects among different nonsynchronous return processes simultaneously. The dynamics of each index return process are marginally depicted by a conventional time series model, which can be obtained by performing many software programs. The joint effects among different indices are captured by adopting a network model with the standardized residual processes of the indices. In particular, the proposed model is capable of effectively accommodating the most updated market information by defining a reasonable adjacency matrix under a network framework. The Granger causality test and Pearson’s correlation test with sharp price movements are employed to determine the lags and significance of the adjacency matrices, respectively.

The NAR-GARCH model is easy to implement, and the model estimation does not require heavy computational costs. It is also convenient to include/withdraw any time series into/from the current model by establishing/removing the marginal model of the related time series, adding/deleting the related rows and columns in the adjacency matrix, and using the LS method to update the coefficients of the network models. Therefore, adaptivity is another advantage of the NAR-GARCH model. Moreover, by applying the NAR-GARCH model to 20 global stock indices during 2006-2020 and comparing its fitting and prediction performances with 3 other commonly used models, the numerical results reveal that the NAR-GARCH model is capable of providing satisfactory market trend predictions and obtaining stable and good investment profits, especially when a market index has strong upward or downward movements.

Usually, a strong upward or downward index movement is caused by a sudden and unexpected event, such as the bankruptcy of Lehman Brothers in 2008 and the COVID-19 outbreak in 2020. When this type of event occurs, some global markets deeply related to the event will have a quick reaction, and other markets may be influenced by the latter event. The proposed NAR-GARCH model is designed to capture this situation effectively. The results of our empirical study provide strong evidence to support that the NAR-GARCH model can depict the above phenomenon well and obtain reliable market trend predictions.

An alternative approach for volatility forecasting is to further include nonlinear patterns, which cannot be captured by GARCH models, in financial time series by the artificial neural network-GARCH (ANN-GARCH) model [21–23]. The ANN-GARCH model aims to forecast multistep price volatility by including many endogenous and exogenous variables as well as GARCH forecast errors to train an ANN model. The numerical results in the literature reveal that the ANN-GARCH model is capable of improving multistep volatility forecasting for time series. Intuitively, if we can improve the 1-step ahead volatility prediction for each index in (3), we might improve the performance of the proposed NAR-GARCH procedure. Nevertheless, the ANN-GARCH model adopts future realized volatilities as the target for multistep volatility forecasting when establishing an ANN model. This approach cannot be applied directly to the prediction of 1-step volatility. In addition, since this type of deep learning framework usually requires more computational costs to train an ANN model than establishing a classical time series model, it is not suitable to apply a rolling-window framework with a fast update frequency, such as the one-day interval used in this study, to investigate its prediction performance. Therefore, further studies are needed to investigate this interesting direction. We refer it to our future research.

## References

[1] Tsay RS. Analysis of Financial Time Series. New Jersey: John Wiley & Sons; 2010.

[2] Nicholson WB, Matteson DS, Bien J. VARX-L: Structured regularization for large vector autoregressions with exogenous variables. International Journal of Forecasting. 2017; 33:627–651. doi: 10.1016/j.ijforecast.2017.01.003

[3] Chu CH, Lo MN, Huang SF, Chen RB. Bayesian structure selection for vector autoregression model. J. Forecasting. 2019;38(5):422–439. doi: 10.1002/for.2573

[4] Bollerslev T. Generalized autoregressive conditional heteroskedasticity. J. Econometrics. 1986;31(3):307–327. doi: 10.1016/0304-4076(86)90063-1

[5] Nelson DB. Conditional heteroskedasticity in asset returns: A new approach. Econometrica. 1991;59:347–370. doi: 10.2307/2938260

[6] Huang SF, Guo MH. Model risk of the implied GARCH-normal model. Quantitative Finance. 2014;14(12):2215–2224. doi: 10.1080/14697688.2011.630323

[7] Huang SF, Lin TY. A linearization of portfolio optimization problem with general risk measures under multivariate conditional heteroskedastic models. Asia-Pacific Journal of Financial Studies. 2018;47(3):449–469. doi: 10.1111/ajfs.12218

[8] Huang SF, Wang DK. A less volatile Value-at-Risk estimation under a semiparametric approach. Working Paper. 2021.

[9] Härdle WK, Okhrin O, Wang W. Hidden Markov structures for dynamic copulae. Econometric Theory. 2015;981–1015.

[10] Grigoryeva L, Ortega JP, Peresetsky A. Volatility forecasting using global stochastic financial trends extracted from non-synchronous data. Econometrics and Statistics. 2018;5:67–82. doi: 10.1016/j.ecosta.2017.01.003

[11] Huang SF, Hsu HL. Prediction intervals for time series and their applications to portfolio selection. REVSTAT-Statistical Journal. 2020;18(1):131–151.

[12] Jondeau E, Rockinger M. The Copula-GARCH model of conditional dependencies: An international stock market application. J. International Money and Finance. 2006;25(5):827–853. doi: 10.1016/j.jimonfin.2006.04.007

[13] Zhu X, Pan R, Li G, Liu Y, Wang H. Network vector autoregression. Annals of Statistics. 2017;45(3):1096–1123.

[14] Granger CWJ. Investigating causal relations by econometric models and cross-spectral methods. Econometrica. 1969;37:424–438. doi: 10.2307/1912791

[15] Engle RF. Autoregressive conditional heteroscedasticity with estimates of the variance of United Kingdom inflation. Econometrica. 1982;50:987–1007. doi: 10.2307/1912773

[16] Fernandez G, Steel MFJ. On Bayesian modeling of fat tails and skewness. J. American Statistical Association. 1998;93:359–371. doi: 10.1080/01621459.1998.10474117

[17] McNeil AJ, Frey R. Estimation of tail-related risk measures for heteroscedastic financial time series: An extreme value approach. J. Empirical Finance. 2000;7(3-4):271–300. doi: 10.1016/S0927-5398(00)00012-8

[18] Kuester K, Mittnik S, Paolella MS. Value-at-Risk prediction: A comparison of alternative strategies. J. Financial Econometrics. 2006;4:53–89.

[19] Cheng WH, Hung JC. Skewness and leptokurtosis in GARCH-typed VaR estimation of petroleum and metal asset returns. J. Empirical Finance. 2011;18(1):160–173. doi: 10.1016/j.jempfin.2010.05.004

[20] Theodossiou P. Financial data and the skewed generalized *t* distribution. Managemet Science. 1998;44(12):1650–1661.

[21] Donaldson RG, Kamstra M. An artificial neural network-GARCH model for international stock return volatility. J. Empirical Finance. 1997;4:17–46. doi: 10.1016/S0927-5398(96)00011-4

[22] Kristjanpoller W, Minutolo MC. Gold price volatility: A forecasting approach using the Artificial Neural Network-GARCH model. Expert Systems. 2015;42:7245–7251. doi: 10.1016/j.eswa.2015.04.058

[23] Kristjanpoller W, Minutolo MC. A hybrid volatility forecasting framework integrating GARCH, artificial neural network, technical analysis and principal components analysis. Expert Systems with Applications. 2018;109:1–11. doi: 10.1016/j.eswa.2018.05.011

